# Malaria, urogenital schistosomiasis and co-infection and nutritional status of school children in Ondo state

**DOI:** 10.1371/journal.pone.0329740

**Published:** 2025-08-08

**Authors:** Esther Mofiyinfoluwa Ola, Temitope Helen Balogun, Rasheed Olayinka Isijola, Oluwaremilekun Grace Ajakaye

**Affiliations:** 1 Department of Animal and Environmental Biology, Adekunle Ajasin University, Akungba Akoko, Ondo State, Nigeria; 2 Laboratory of Molecular Parasitology and Genomics of Neglected Tropical Diseases, Adekunle Ajasin University, Akungba-Akoko, Ondo State, Nigeria; University of Uyo, NIGERIA

## Abstract

Parasitic infections are a major cause of morbidity and mortality in Nigeria, with malaria and schistosomiasis having the highest burden. This study investigated the prevalence of malaria, urogenital schistosomiasis, and co-infections and their impact on the nutritional status of schoolchildren in two communities in Ondo State. A total of 185 participants from Ipogun and Oke Igbo were screened for malaria and schistosomiasis infection using the ParaHit malaria rapid diagnostic test kit and urine microscopy. Anthropometric measurements were used to assess the nutritional status of the participants. In this study, a higher prevalence of malaria was recorded in Oke Igbo, with 36 individuals (57.1%), compared to 60 individuals (49.2%) in Ipogun. Urogenital schistosomiasis was also more prevalent in Oke Igbo, affecting 18 individuals (28.6%), while only 5 individuals (4.1%) were affected in Ipogun. Co-infection with both diseases was more common in Oke Igbo, with 13 cases (20.6%), compared to 4 cases (3.3%) in Ipogun. However, malnutrition rates were similar between the two communities, with 60 cases (77.9%) in Ipogun and 28 cases (75.5%) in Oke Igbo. Notably, participants with either malaria or urogenital schistosomiasis, as well as those co-infected, exhibited a higher frequency of chronic malnutrition. The likelihood of co-infection was significantly associated with gender and locality, with individuals in Oke Igbo being 0.78 times less likely to be co-infected (P = 0.00; CI = 0.09–0.49), while males were 2.19 times more likely to have co-infections (P = 0.02; CI = 1.13–4.28). This study emphasised the significant health burden posed by malaria and urogenital schistosomiasis co-infections among schoolchildren in Ondo State, highlighting the need for comprehensive health and nutritional interventions to address the challenges associated with these parasitic diseases.

## Background

Malaria and schistosomiasis are major public health challenges and are the leading parasitic illnesses responsible for significant morbidity and mortality worldwide [1,2]. In sub-Saharan Africa (SSA), where over 90% of these diseases manifest, co-infections with these parasites are commonly reported [3]. In Nigeria, *Plasmodium falciparum* and *P. vivax* are the primary etiological agents of malaria [4]. In 2022, severe malaria caused approximately 194,000 deaths in Nigeria, with about 80% of these fatalities occurring in children under five, who account for 39% of all malaria deaths in this age group globally [5]. According to [6], Ondo State is one of the highest contributors to malaria cases in Nigeria, with an estimated 1.7 million cases. Malaria parasites are transmitted to humans by the bite of infected female Anopheles mosquitoes during blood meals. Once inside the body, the parasites develop in hepatocytes, producing merozoites that invade red blood cells [7]. The repeated infections occurring mostly in childhood impose significant burdens on households, health services, and the economic development of communities and nations [8].

On the other hand, schistosomiasis is a neglected tropical disease caused by the trematode blood flukes of the genus *Schistosoma*. Schistosomes are digenetic intravascular parasites that live in the venous portal mesenteric system of their definitive hosts [9]. *S. manson*i and *S. japonicum* are responsible for the intestinal forms of schistosomiasis, while *S. haematobium* causes urogenital schistosomiasis (UgS) [10]. Nigeria has the highest prevalence of schistosomiasis worldwide, with over 20 million cases, primarily from *S. haematobium*, and schoolchildren are the most vulnerable group [11]. The World Health Organisation (WHO) reports that schistosomiasis in Nigeria led to 2.5 million disability-adjusted life years lost and approximately 24,000 annual mortalities [12]. Freshwater snails of the genus *Bulinus* serve as intermediate hosts in the life cycle of *S. haematobium*, releasing cercariae into freshwater, which humans can contract through contact with infected water bodies [13]. UgS affects the urinary tracts and genital organs, resulting in haematuria, bladder disease, and long-term reproductive difficulties [14].

Both schistosomiasis and malaria have been linked, separately and together, to nutritional stress in infected individuals. Children with co-infections often experience poor growth and cognitive performance [15]. Research indicates that even subclinical infections, regardless of severity, increase nutrient losses and reduce nutrient intake. Parasitic infections can disrupt absorptive surfaces, physically restrict the gastrointestinal lumen, produce proteolytic chemicals, and consume vital nutrients, ultimately diminishing the body’s nutrient availability [16]. Children are especially susceptible to the effects of parasitic diseases because of their heightened susceptibility to malnutrition. Malnutrition and recurrent illnesses can cause excess morbidity among impoverished populations in developing nations, a cycle that can last for generations [16].

Although previous studies have shown that malaria and urinary schistosomiasis are prevalent in Ondo State, few have explored the relationship between malaria and schistosomiasis co-infection and malnutrition [17–18]. School children constitute an ideal target group for investigation of both diseases because of their heightened susceptibility to parasitic infections and increased risk of malnutrition [19]. This study, therefore, investigated the prevalence of malaria, urogenital schistosomiasis, and co-infections, as well as their impact on the nutritional status of schoolchildren in Ondo State.

## Methods

### Ethical considerations

Ethical permission to conduct this study was approved by the Ondo State Ministry of Health (OSHREC 23/11/2023/598). Participants for this study were recruited between March and April, 2024. Informed written consent was obtained from the parents or guardians of the participants. Additionally, for children who were ≥ 10 years, an additional verbal assent was obtained in front of class teachers. Praziquantel was administered to all participants of the study according to the WHO recommendations.

### Study sites

**Fig 1:**
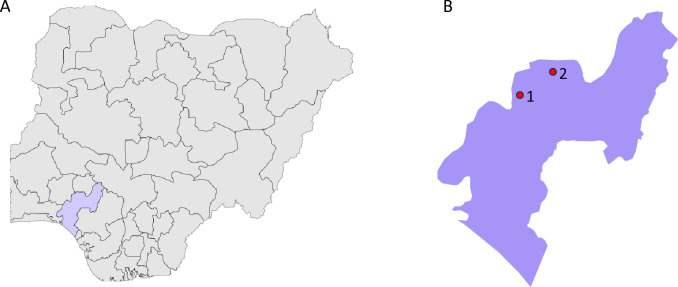
Map of Study Sites. **(A)** Map of Nigeria highlighting Ondo State. **(B)** Map of Ondo state showing Ile Oluji/Oke Igbo [1] and Ifedore [2] LGAs.Sample size determination.

This study was conducted in the Ipogun and Oke Igbo communities in Ondo State, Nigeria (Fig 1). These study sites were selected due to their long-standing history of schistosomiasis endemicity [20–24]. Ipogun is located in the Ifedore Local Government Area (LGA) of Ondo State, about 17 km from the state capital. It lies at a longitude of 7.3632° N and a latitude of 5.0883° E, with a land area of 295 square kilometres. The area has a growing population of 176,327 as per the 2006 census [25]. Ipogun has a tropical savanna climate with an annual average temperature of approximately 25.1°C and a high humidity level of 80% [26]. The prominent rivers in the community are the Aponmu and Ogun rivers [27]. Neighbouring towns include Ilara-mokin, Ibule-soro, Itaoniyan, Igbara-oke, etc. [28].

Oke Igbo is located in Ile-Oluji/Oke Igbo LGA, lying at a longitude of 7.5756°N and a latitude of 4.8125°E, with a land area of 698 km^2^ and a population of 172,870 at the 2006 census [25]. It has a mean annual temperature of 25°C and a high humidity level of 80%. The average annual precipitation is about 1600–2000 mm. Humidity is relatively high for a good part of the year [26]. The most common rivers in this community are the Aigo, Olori, and Ojege rivers [27].

Based on previous studies [5,29], the sample size for this study was determined using the single population proportion formula n = Z^2^P(1-P)/d2, with a 95% confidence level and a precision of 0.05. A total of 185 participants were recruited, consisting of 122 from Oke Igbo and 63 from Ipogun. Participants were schoolchildren between four and sixteen years. Informed consent was obtained from parents and guardians after explaining the study’s objectives, and participation was entirely voluntary.

### Collection of sociodemographic and anthropometric data

Anthropometric parameters, including weight and height, were measured using a weighing scale and measuring tape, respectively. Sociodemographic characteristics such as age and sex were collected alongside these measurements. Data were documented on a predesigned epidemiological form and manually entered into Microsoft Excel and cross-checked for errors before being saved on a password-protected laptop. Unique identifiers were used to deidentify all collected data.

### Malaria diagnosis

Each participant was tested for malaria parasite infection using the ParaHit® ver 1.0 Rapid Diagnostic Test (RDT) for *P. falciparum* (lot number: 4000028593). The test kit included a cassette, lancet, pipette, and swab. The tests were carried out according to the manufacturer’s instructions. Briefly, the thumb for the procedure was cleaned with a sterile wipe, after which it was punctured with a sterile lancet. Approximately 8µl of blood was collected with a micropipette and transferred to the test device. Following the addition of 200µl of buffer solution, results were read between 20 and 30 minutes. The appearance of two red-coloured bands each at the test (T) and control (C) regions indicated the presence of *P. falciparum* antigen, while a single band in the control region indicated absence. Tests were considered invalid when no band appeared in the control region.

### Urine microscopy for diagnosis of UgS

Urine samples were collected in sterile, well-labelled 25 ml screw-cap plastic containers. After transportation to the laboratory, samples were allowed to settle for two hours. Excess urine was carefully decanted, and up to 10 ml of the remaining sedimented sample was gradually pipetted onto a small petri dish [30] for examination under a microscope to detect *S. haematobium* eggs [31].

### Data analysis

Demographic data were analysed using Microsoft Excel (Version 2407), and chi-square tests were used to calculate the p-values. P values less than 0.05 were considered significant. The mean and standard deviation for age, weight, and height were calculated as described by [32]. Risk factors for malnutrition and coinfection were analysed using logistic regression in R. Nutritional indices, including height-for-age (stunting), weight-for-height (wasting), and weight-for-age (underweight), were calculated using the Anthro WHO software. According to WHO standards, children scoring < −2 SD in at least one anthropometric index were classified as moderately malnourished, while those scoring < −3 SD were considered severely malnourished [33–34].

## Results

### Socio-demographic and nutritional characteristics of the study population

A total of one hundred and eighty-five (185) volunteers were screened to assess their nutritional status and detect the presence of *P. falciparum* and *S. haematobium* infections. In Ipogun, the participants consisted of 51.6% males and 48.4% females. The mean age was 8.6 years (SD = 2.8), ranging from 3 to 15 years. The mean height was 119.4 cm (SD = 15.8), with values ranging from 78 cm to 153 cm, while the mean weight was 23.9 kg (SD = 7.7), ranging from 10.2 kg to 73.4 kg. Nutritional assessments indicated that 9 (52.9%) of participants experienced wasting and 58 (79.5%) were stunted, while 43 (47.8%) were underweight and 60 (77.9%) were malnourished.

In Oke Igbo, out of the sixty-three (63) volunteers, 42.9% were males and 57.1% were females. The mean age was 9.3 years (SD = 4.1), with ages ranging from 2 to 18 years. The mean height was 115.3 cm (SD = 18.4), with a range of 70–150 cm. The mean weight was 22.8 kg (SD = 8.3), ranging from 8.1 kg to 47.7 kg (Table 1). Nutritional indices revealed that 2 (18.2%) of participants had wasting, 18 (69.2%) were stunted, 19 (51.4%) were underweight, and 28 (75.7%) were malnourished.

**Table 1:** Socio-demographic and nutritional characteristics of the study population.

Location		
	Ipogun (N = 122)	Oke Igbo (N = 63)
**Gender**	**Frequency n (%)**	
Male	63 (51.6)	27 (42.9)
Female	59 (48.4)	36 (57.1)
**Age (years)**		
Mean (SD)	8.6 (2.8)	9.3 (4.1)
Min	3	2
Max	15	16
**Height (cm)**		
Mean (SD)	119.4 (15.8)	115.3 (18.4)
Min	78	70
Max	153	150
**Weight (kg)**		
Mean (SD)	23.9 (7.7)	22.8 (8.3)
Min	10.2	8.1
Max **Nutritional indices (Age group)**	73.4	47.7
Wasting (< 5 years)	9 (52.9)	2 (18.2)
Stunting (5–10 years)	58 (79.5)	18 (69.2)
Underweight (≤ 10 years)	43 (47.8)	19 (51.4)
Malnutrition (≤ 10 years)	60 (77.9)	28 (75.7)

### Prevalence of malaria in the study population

In Ipogun, the highest prevalence of *P. falciparum* infection was observed among children aged 11–15 years (65.6%), followed by those aged 6–10 years (45.1%) and 0–5 years (36.8%). Males had a slightly higher infection rate (55.6%) compared to females (49.4%). In Oke Igbo, malaria prevalence was highest among the 6–10 (68.2%) and 11–15 (54.2%) age groups, followed by the 16–20 (50.0%) and 0–5 (46.7%) age groups. Interestingly, females in Oke Igbo showed a higher prevalence (61.1%) compared to males (51.9%). However, no statistically significant differences in malaria prevalence were observed across age groups or between sexes in either community (*p* > 0.05) (Table 2).

**Table 2:** Prevalence of malaria in Ipogun and Oke Igbo.

Ipogun				Oke Igbo		
Parameter	Total n (%)	Infected n (%)	P value	Total n (%)	Infected n (%)	P value
**Age**			0.53			0.92
0-5	19 (15.6)	7 (36.8)		15 (23.8)	7 (46.7)	
6-10	71 (58.2)	32 (45.1)		22 (34.9)	15 (68.2)	
11-15	32 (26.2)	21 (65.6)		24 (38.1)	13 (54.2)	
16-20	0 (0.0)	0 (0.0)		2 (3.2)	1 (50.0)	
**Gender**			0.39			0.70
Male	63 (51.6)	35 (55.6)		27 (42.9)	14 (51.9)	
Female	59 (48.4)	25 (49.4)		36 (57.1)	22 (61.1)	

### Prevalence of urogenital schistosomiasis in the study population

In Ipogun, the highest prevalence of UgS was observed among participants aged 11–15 years (6.3%), followed by those aged 6–10 years (4.2%). Males had a slightly higher prevalence (4.8%) compared to females (3.4%). In Oke Igbo, UgS infection was most prevalent among the 11–15 age group (37.5%), followed by the 0–5 (26.7%) and 6–10 (22.7%) age groups. No infections were recorded among individuals aged 16–20 years. Male participants in Oke Igbo had a significantly higher prevalence (40.7%) compared to females (19.4%). However, no statistically significant differences in UgS prevalence were observed between sexes or across age groups in either community (*p* > 0.05) (Table 3).

**Table 3:** Prevalence of urogenital schistosomiasis in Ipogun and Oke Igbo.

Ipogun				Oke Igbo		
Parameter	Total n (%)	Infected n (%)	P value	Total n (%)	Infected n (%)	P value
**Age**			0.54			0.55
0-5	19 (15.6)	0 (0.0)		15 (23.8)	4 (26.7)	
6-10	71 (58.2)	3 (4.2)		22 (34.9)	5 (22.7)	
11-15	32 (26.2)	2 (6.3)		24 (38.1)	9 (37.5)	
16-20	0 (0.0)	0 (0.0)		2 (3.2)	0 (0.0)	
**Gender**			0.70			0.06
Male	63 (51.6)	3 (4.8)		27 (42.9)	11 (40.7)	
Female	59 (48.4)	2 (3.4)		36 (57.1)	7 (19.4)	

### Co-infection of malaria and UgS based on sociodemographic factors

In Ipogun, co-infection was also more common in the age groups 11–15 years (6.3%) and 6–10 years (2.8%), while there was no co-infected individual in the 0–5 years age group. Similarly, a higher proportion of males (4.8%) were co-infected compared to females (1.7%). In Oke Igbo, malaria and UgS co-infection were more prevalent among age groups 11–15 years (29.2%) compared to age groups 6–10 years (18.2%) and 0–5 years (13.3%). The results also indicated that a higher percentage of males (25.9%) were co-infected than females (16.7%).

Statistical analysis revealed no significant difference in co-infection prevalence based on age or sex within these groups (P > 0.05) (Table 4).

**Table 4:** Co-infection of malaria and UgS based on socio-demographic factors.

Ipogun (N = 122)				Oke Igbo (N = 63)		
Parameter	Total n (%)	Co-infectivity n (%)	P value	Total n (%)	Co-infectivity n (%)	P value
**Age**			0.45			0.54
0-5	19 (15.6)	0 (0.0)		15 (23.8)	2 (13.3)	
6-10	71 (58.2)	2 (2.8)		22 (34.9)	4 (18.2)	
11-15	32 (26.2)	2 (6.3)		24 (38.1)	7 (29.2)	
16-20	0 (0.0)	0 (0.0)		2 (3.2)	0 (0.0)	
**Gender**			0.34			0.37
Male	63 (51.6)	3 (4.8)		27 (42.9)	7 (25.9)	
Female	59 (48.4)	1 (1.7)		36 (57.1)	6 (16.7)	

### Burden of malnutrition in relation to malaria, UgS, and co-infection

In Ipogun, malaria infection was detected in 49.2% of the total population. Among those infected, 90.0% were classified as normal, 1.7% as moderately malnourished, and 8.3% as chronically malnourished. The prevalence of urinary schistosomiasis (UgS) was 4.1%, with 40.0% of infections occurring in non-malnourished individuals and 60.0% in those suffering from chronic malnutrition. Co-infection with malaria and UgS was found in 3.3% of the population, including 25.0% of normal cases and 75.0% of chronically malnourished individuals. There was a significant difference between disease occurrence and malnutrition.

In Oke Igbo, the burden of malaria was higher, affecting 57.11% of the total population. Among those infected, 41.7% were normal, 13.9% were moderately malnourished, and 44.4% were chronically malnourished. UgS infection also showed a higher prevalence in Oke Igbo, affecting 28.6% of participants, with 44.4% classified as normal, 11.2% as moderately malnourished, and 44.4% as chronically malnourished. Co-infection with malaria and UgS occurred in 20.6% of the population, with 38.5% normal cases, 7.7% moderately malnourished, and 53.8% chronically malnourished individuals. The observed differences between disease occurrence and malnutrition were statistically insignificant in Oke Igbo (Table 5).

**Table 5:** Burden of malnutrition in relation to malaria, UgS and co-infection.

Ipogun (N = 122)						Oke Igbo (N = 63)				
	Infected n (%)	N n (%)	MM n (%)	SM n (%)	P value	Infected n (%)	N n (%)	MM n (%)	SM n (%)	P value
**Infection**					0.00					0.96
Malaria*	60 (49.2)	54 (90.0)	1 (1.7)	5 (8.3)		36 (57.1)	15 (41.7)	5 (13.9)	16 (44.4)	
**UgS**	5 (4.1)	2 (40.0)	0 (0.0)	3 (60.0)		18 (28.6)	8 (44.4)	2 (11.2)	8 (44.4)	
**Co-infection**	4 (3.3)	1 (25.0)	0 (0.0)	3 (75.0)		13 (20.6)	5 (38.5)	1 (7.7)	7 (53.8)	

*P value was significant for infection between both sites (p = 0.00).

N = normal, MM = moderate malnutrition (<−2SD), CM = severe malnutrition (<−3SD). P values were calculated based on comparison between disease prevalence and malnutrition categories for each study location.

### Risk factors affecting malnutrition

Table 6 presents the results of the logistic regression analysis assessing risk factors associated with malnutrition. None of the examined risk factors showed a statistically significant association with malnutrition (p > 0.05). Similarly, the separate regression analyses for wasting, stunting, and underweight did not identify any significant associations between the individual nutritional indices and the risk factors analysed (Tables 1–3 in S1 Table).

**Table 6:** Risk factors affecting malnutrition.

Parameter	aOR	P value	CI (95%)
**Location**			
Ipogun			
Oke Igbo	0.52	0.28	0.16-1.72
**Gender**			
Female			
Male	1.11	0.81	0.49-2.51
**Age category**			
Over 5			
Under 5	0.64	0.37	0.24-1.80
**Malaria infectivity**			
Negative			
Positive	1.40	0.61	0.39-5.36
**UgS infectivity**			
Negative			
Positive	1.49	0.64	0.31-8.88
**Co-infectivity**			
Negative			
Positive	0.56	0.21	0.22-1.38

aOR = adjusted Odds Ratio, CI = Confidence Interval

### Risk factors affecting malaria and UgS co-infection

The logistic regression analysis of various sociodemographic and clinical parameters showed that location and gender were risk factors affecting malaria and UgS coinfection. Compared to Ipogun, the reference group, Oke Igbo had a significant odds ratio (aOR) of 0.22, indicating that the odds of coinfection in Oke Igbo are 78% lower compared to Ipogun. The odds of coinfection in males were also 2.19 times higher than in females, with an odds ratio (aOR) of 2.19. Although age and malnutrition status also lowered the odds of co-infection, they were statistically insignificant (p > 0.05) (Table 7).

**Table 7:** Risk factors affecting malaria and UgS co-infection.

Parameter	aOR	P value	CI (95%)
**Location**			
Ipogun			
Oke Igbo	0.22	0.00^*^	0.09-0.49
**Gender**			
Female			
Male	2.19	0.02^*^	1.13-4.28
**Age category**			
Over 5			
Under 5	0.59	0.28	0.22-1.51
**Malnutrition**			
Normal			
Moderate	0.57	0.26	0.21-1.52
Severe	0.66	0.35	0.27-1.59

*P < 0.05, aOR = adjusted Odds Ratio, CI = Confidence Interval

## Discussion

This study found that *falciparum* malaria and urogenital schistosomiasis co-infection are of public health importance among school children with potential impact on their nutritional status.

The prevalence of *P. falciparum* in Oke Igbo (57.1%) and Ipogun (49.2%) showed that both areas are malaria endemic. This may be due to the rural nature of the study areas, which provide ideal opportunities such as stagnant water bodies, poor drainage systems, and bushy environments for the breeding of *Anopheles* mosquitoes [35]. The observed prevalence was, however, lower than the 67.6%, 80.2%, and 86.0% observations of Simon-Oke *et al.*, Omoya and Ajayi, and Enitan *et al*. [36–38], respectively, in Ondo State. In both study sites, there was no significant variation between gender and malaria infection. However, gender-related disparities have been reported in Ondo State [37,39–41]. Although children under five are the most at risk of malaria, school-aged children are also highly susceptible, with up to 70% potentially carrying asymptomatic parasitemia in high-transmission areas [42]. Although the observed variation in infection frequency across age groups was not statistically significant, it may still reflect underlying factors such as poor immunity, lack of protective measures, outdoor exposure, inadequate health services, socio-economic conditions, and favourable mosquito habitats [43–44].

In this study, the 4.1% prevalence of UgS in Ipogun was lower than the prevalence rate recorded in the same community in previous years by [45] and [46], which may be due to the annual administration of praziquantel in the area [46]. Also, the community is often sampled for schistosomiasis, which might have increased the knowledge of the disease in the area. Oke Igbo had a 28.6% prevalence of UgS, which was higher than the prevalence rate [20] found in the same community in previous years. This could be due to constant reinfection as a result of water contact activities. The age- and gender-related patterns observed in our study, though not statistically significant, are consistent with the known epidemiology of urogenital schistosomiasis and may be attributed to frequent water contact activities. Similar trends have been reported in other endemic areas within Ondo State [47–48]. We also recorded a 3.3% and 20.6% malaria and UgS co-infection in Ipogun and Oke Igbo respectively. These findings are dissimilar to that of [49], who reported a 6% prevalence of malaria and UgS co-infection in Ibadan and Akure in 2012. The findings of this survey also disagree with those of [50,51], and [52], who reported higher prevalences in North Central Nigeria, Ogun, and River State respectively. The observed prevalences of malaria and UgS co-infection in this study could be attributed to several factors such as awareness, mass drug administration, level of urbanisation and seasonal variations. Various factors such as cultural practices, environmental factors, limited availability of basic amenities, and insufficient disease prevention programs could contribute to disease coinfections in endemic settings [53].

The prevalence of co-infection in the study areas was higher among children aged 11–15 years. This observation contrasts the findings by [50–52], and [54]. In terms of gender, males in both study locations experienced a higher frequency of co-infection, which agrees with [54] but disagrees with [50] and [51], who found higher co-infectivity among females. Males and those aged 11–15 are often more physically active and curious, spending more time outdoors and interacting with their environment compared to younger children. This increased mobility and exploration can lead to more frequent exposure to mosquito-prone areas and infested water bodies. They may also have more responsibilities in helping with family activities, such as tending to farms or livestock, which might bring them closer to water sources where both vectors are prevalent [55].

In this study, we observed a 51.4% prevalence of underweight in Oke Igbo, which was slightly higher than that in Ipogun (47.8%). The observed prevalence was higher than the report by [56] and [57] in Ebonyi and Enugu respectively. Cultural dietary practices and limited access to nutrient-rich foods, combined with the disease burden and limited availability of healthcare facilities, could likely explain the higher underweight problems in the study population.

The prevalence of stunting in Ipogun (79.5%) and Oke Igbo (69.2%) was similar to the study by [58] in Kaduna but lower than other reports from different parts of Nigeria [59–61]. However, it was higher than the 2018 national prevalence of stunting in Nigeria (37%), which was classified as critical by [33]. The high prevalence of stunting in the study areas could be indicative of the long-term, cumulative effects of inadequate nutrition and poor health status [62]. Socioeconomic factors such as poverty, lack of education, and limited access to healthcare might have contributed to the high stunting rates.

There was a disparity in the prevalence of wasting in the study areas (Ipogun, 52.9% and Oke Igbo, 18.2%). Compared to reports from different parts of the country [63–66], the observation in Ipogun was higher, while that of Oke Igbo was lower. The disparity in the burden of wasting between the two study areas and, in comparison, to other regions highlights the impact of local factors such as cultural and dietary practices, health, and socio-economic conditions on nutritional status [67].

The prevalence of chronic malnutrition among individuals infected with malaria was significantly higher in Oke Igbo (44.4%) compared to Ipogun (8.3%). Previous studies found mixed results regarding the effect of malaria on growth and malnutrition [68–72]. Frequent recurrence of malaria, common in regions with high transmission rates, can lead to prolonged inflammation and poor appetite, worsening nutritional deficits and leading to chronic malnutrition [73]. This study also observed a high prevalence of chronic malnutrition among persons affected with UgS in both study areas. *Schistosoma* infection affects nutrient absorption, particularly in the intestines, leading to impaired digestion and poor absorption of essential vitamins and minerals [74–76]. Protein loss and fibrosis can further increase malnutrition [77]. Our observation could be due to the fact that repeated cycles of infection and reinfection observed over the years in both areas have potentially resulted in sustained nutritional depletion and chronic malnutrition [78]. Although our study recorded a low prevalence of coinfection with malnutrition, the interactions between coinfections and malnutrition cannot be downplayed. The high occurrence of chronic malnutrition among coinfected individuals which was significantly high in Oke Igbo suggests that both diseases may negatively impact the nutritional status of children in the area. Similar observations have been made by Dassah *et al*. [79]. This could be because schistosomiasis and malaria impair immunity, increase energy requirements, block nutrient absorption, and drain nutrients [73–77]. The situation is worsened by the lack of food and healthcare in these areas [15]. The small number of coinfected individuals at both sites, however, limits the ability to confidently detect disease associations with malnutrition. Consequently, the observed pattern may not be generalizable, and larger sample sizes are necessary to draw reliable conclusions about the relationship between coinfection and malnutrition.

Despite the high prevalence of wasting, underweight, stunting, and malnutrition in this study, no risk factor was found to be significantly associated. This suggests there could be other potential non-disease-related factors contributing to malnutrition in the study populations. Duru *et al*. [80] reported a direct association between childhood malnutrition and low maternal education, linking this to broader issues such as limited socioeconomic advancement, restricted access to healthcare, and reduced maternal empowerment. Similarly, Obasohan *et al*. [81] identified several determinants of child health, including age, gender, birth weight, birth type, and place of delivery.

We also observed that living in Oke Igbo and being male were significant risk factors for co-infection with malaria and UgS. This is likely due to environmental, cultural, and behavioural factors. In conclusion, this study highlights the substantial public health burden of *P. falciparum* malaria, urogenital schistosomiasis, and their co-infection among school-aged children. The observed high prevalence of malnutrition further underscores the need for additional research into its underlying causes, including non-disease-related factors.

## Supporting information

S1 Table Risk factors affecting stunting, underweight and wasting. (XLSX)
